# Pleiotropic effects of the COX-2/PGE2 axis in the glioblastoma tumor microenvironment

**DOI:** 10.3389/fonc.2022.1116014

**Published:** 2023-01-26

**Authors:** Phillip T. Dean, Shelley B. Hooks

**Affiliations:** Department of Pharmaceutical and Biomedical Sciences, College of Pharmacy, University of Georgia, Athens, GA, United States

**Keywords:** glioblastoma, COX-2, PGE2, microglia, macrophage, inflammation, tumor microenvironment, cancer

## Abstract

Glioblastoma (GBM) is the most common and aggressive form of malignant glioma. The GBM tumor microenvironment (TME) is a complex ecosystem of heterogeneous cells and signaling factors. Glioma associated macrophages and microglia (GAMs) constitute a significant portion of the TME, suggesting that their functional attributes play a crucial role in cancer homeostasis. In GBM, an elevated GAM population is associated with poor prognosis and therapeutic resistance. Neoplastic cells recruit these myeloid populations through release of chemoattractant factors and dysregulate their induction of inflammatory programs. GAMs become protumoral advocates through production a variety of cytokines, inflammatory mediators, and growth factors that can drive cancer proliferation, invasion, immune evasion, and angiogenesis. Among these inflammatory factors, cyclooxygenase-2 (COX-2) and its downstream product, prostaglandin E2 (PGE2), are highly enriched in GBM and their overexpression is positively correlated with poor prognosis in patients. Both tumor cells and GAMs have the ability to signal through the COX-2 PGE2 axis and respond in an autocrine/paracrine manner. In the GBM TME, enhanced signaling through the COX-2/PGE2 axis leads to pleotropic effects that impact GAM dynamics and drive tumor progression.

## Introduction

Glioblastoma multiforme (GBM) is the most common and aggressive form of central nervous system (CNS) tumor. GBM accounts for 48.3% of all malignant brain tumors. GBM patients have a median survival rate of only 14-17 months with standard treatment including surgical resection, chemotherapy, and radiotherapy, and a median survival of less than 6 months without therapeutic intervention (1–3). Poor prognosis in GBM patients is linked to high intra-and inter-tumor heterogeneity, chemoresistance, and an immunosuppressive environment (4). The GBM tumor microenvironment (TME) plays a crucial role in development and progression of the disease. The TME is a dynamic cellular and molecular ecosystem of tumor cells, glioblastoma stem cells (GSCs), stromal cells (fibroblasts, endothelial), and immune cells (microglia, macrophages, T-cells, B-Cells) actively responding to their surrounding cells, tissues, and molecular cues (5, 6). This highly complex network communicates through production of cytokines, chemokines, bioactive lipids, and extracellular matrix components. Together they dictate diverse pro-inflammatory and anti-inflammatory responses that shape their environment through communication and interaction (7).

The most abundant and multifaceted members of the GBM-TME are the glioma associated microglia and peripheral macrophages (GAMs). They constitute up to 30-50% of tumor associated cells, and thus have a strong influence on the GBM-TME (8, 9). The degree of GAM integration in the GBM-TME is positively correlated with tumor grade and inversely correlated with patient survival (7, 10). The presence of these inflammatory cells in the TME leads to dysregulated inflammation and plays a key role in the immunosuppressive nature of GBM, consistent with the well-established association between cancer and inflammation (11). In contrast to their phagocytic and cytotoxic capabilities against infection, GAMs produce inflammatory mediators that promote tumor growth, immunosuppression, and angiogenesis. GAMs produce an array of cytokines, growth factors, and bioactive lipids that aid in a pro-tumoral shift, such as Interleukin (IL)-1β, IL-6, transforming growth factor-β (TGF-β), epidermal growth factor (EGF), and the prostaglandin E2 (PGE2) (12, 13). PGE2 is highly enriched in the GBM-TME and has a substantial impact on proliferation, migration, immunosuppression, and angiogenesis. Similarly, cyclooxygenase 2 (COX-2), the enzyme responsible for PGE2 production, is also highly upregulated in GBM and is associated with tumor growth (Shono et al., no date; 14–16). Here, we review the current understanding of the COX-2/PGE2 signaling axis in GAMs, its regulation of the tumor microenvironment, and its impact on GBM tumor progression.

Inflammation is well established as a robust driver of cancer and is now considered to be one of the hallmarks of cancer (11). In natural inflammatory responses, infections and cell damage are cleared by immune cells that launch an acute proinflammatory response to neutralize the threat. Once the threat has been neutralized, immune cells launch an anti-inflammatory response to resolve inflammation. In aberrant situations, such as chronic inflammation and cancer, the threat may not be neutralized, causing dysregulation of the inflammatory program. The relationship between glioma and inflammation is characterized by multiple key steps: first, the recruitment and infiltration of immune cells to the site of the tumor; second, complex signaling crosstalk between the tumor cells and multiple types of immune cells mediated through small molecule release and activation of receptors on neighboring cells; third, tumor cell responses including proliferation, transcriptional regulation, migration, and differentiation; and finally, tumor progression driven by immune evasion, neovascularization, and tissue remodeling. Activation of GAMs induces the release of cytokines, growth factors, and other inflammatory mediators that promote tumor growth, angiogenesis, and an immunosuppressed state.

## Microglia and macrophages in the CNS

Microglia and brain infiltrating macrophages serve an essential role as immune sentinels, responding to infection and injury in the central nervous system (CNS) to maintain brain homeostasis (5, 17). Bone marrow derived macrophages (BMDMs) originate in the bone marrow as peripheral hematopoietic progenitors, and they become circulating monocytes in the blood stream. BMDMs are highly motile as they locate to target tissue, but motility lowers as they approach a tumor and eventually take residence in the tumor tissue. Microglia, found throughout the brain, represent a distinct myeloid population, and are considered the resident macrophages of the CNS. Microglia are primarily derived from erythro-myeloid progenitor cells in the yolk sac during early embryogenesis and are long lived, relying on self-renewal in the CNS (18, 19). They maintain homeostatic conditions by supporting neurogenesis, synaptic pruning, and phagocytotic clearing of apoptotic cells and debris (20, 21). Microglia exhibit diverse morphologies and phenotypes in response to various stimuli. Surveilling microglia are highly ramified to efficiently respond to environmental stimuli (22). Once activated, they rapidly change to an amoeboid morphology (23, 24). Microglia activation leads to production of IL-1β, which plays an important role in modulating the blood brain barrier (BBB) and promotes a leaky state that allows entrance of bone marrow derived immune cells to enter the brain (25). BMDMs have remarkably similar morphology to the ameboid shaped microglia making it challenging to distinguish between the two histologically (19). Thus, both BMDM-derived brain infiltrating macrophages and resident microglia are present in the brain and in the GBM-TME, and these cells can be functionally and phenotypically difficult to distinguish. Compared to BMDMs, microglia have limited migratory capacity and instead use their processes to extend and retract, constantly surveilling their surroundings (26). These migratory differences lead to the differential distributions between macrophages and microglia in the GBM-TME. Single-cell RNAseq analysis of GBM revealed that highly motile infiltrating macrophages were primarily located in the central regions of the tumor while microglia tend to surround the outer edge of the tumor (27). Additionally, GBM tumors typically display necrotic cores and microvascular hyperplasia due to the hypoxic environment. GAMs accumulate in these hypoxic/necrotic areas of tumors where they support tumor proliferation and angiogenesis (28, 29).

## GAMS in the GBM microenvironment

In glioma, macrophages and microglia are recruited to the tumor site by glioma-derived chemoattractant factors such as colony stimulating factor 1 (CSF1), C-C motif chemokine ligand 2 (CCL2; also known as monocyte chemoattractant protein 1, MCP-1), fractalkine (CX3CL1), and vascular endothelial growth factor (VEGF) (30–33). Following recruitment, GAMs secrete inflammatory mediators that regulate angiogenesis, proliferation, and immunosuppression in the GBM-TME. PGE2 is emerging as a key mediator of these effects, and both PGE2 and its upstream biosynthetic enzyme COX-2 are overexpressed in the GBM-TME, are associated with poor prognosis, and mediate pleiotropic effects that support glioma proliferation, angiogenesis, and immunosuppression (16, 34).

GBM tumors are highly vascular and rely on neovascularization for tumor growth. Microglia and macrophages play a supporting role in this process through the production of angiogenic factors and degradation of the extracellular matrix (ECM) (35). Depletion of microglia and macrophages in an animal model of GBM resulted in reduced micro-vessel density (MVD), proliferation, and overall tumor volume (36). Additionally, selective depletion of only microglia led to a comparable attenuation of MVD to that of total GAM depletion, suggesting that microglia are particularly important immune facilitators of angiogenesis in glioma (36). GAMs release multiple angiogenic factors that promote angiogenesis and invasiveness, including transforming growth factor β (TGF-β), IL-6, and vascular endothelial growth factor (VEGF) (37). VEGF expression is upregulated in hypoxic regions where it acts as a robust chemoattractant to recruit GAMs, which in turn promote angiogenesis (29). In the presence of glioma cells, microglia produce significant amounts of TGF-β, which in turn induces production of matrix metalloproteinase 9 (MMP9) and MMP2, leading to degradation of ECM and supporting glioma stem cell invasion (38). GSCs are treatment resistant, multipotent, self-renewing cells with high heterogeneity (39, 40). GAMs and GSCs are often functionally interconnected and co-localized. Mapping of cellular distribution in human GBM revealed that striking numbers of GAMs were located around GSC clusters and, as observed with GAMS, the density of GSCs positively correlated to tumor grade (40). GAMs accumulate in perivascular regions where they produce proangiogenic factors such as VEGF and CXCL2, due to chemoattractant release from GSCs (36). Taken together, these observations suggest a complex signaling interplay between tumor cells, stem cells, and GAMs to regulate angiogenesis and invasion. Growing evidence suggests that COX-2 and PGE2 are key mediators of the effect of GAMs on angiogenesis. COX-2 and PGE2 are produced by microglia and macrophages, and PGE2 accumulation is particularly high in hypoxic/necrotic regions of the TME (16, 41). PGE2 in the TME is linked to increased expression of glioma-derived monocyte chemoattractant CCL2/MCP-1, leading to active recruitment of GAMs (31, 42). In response, GAMs induce IL-6 production, which increases GBM invasiveness (31). COX-2 and PGE2 regulate expression of VEGF and trigger increased MVD, suggesting that this pathway is critical to the signaling networks that regulate angiogenesis in the GBM-TME (15).

Growing evidence suggests that GAMs also play a key role in establishing the immunosuppressant microenvironment that is characteristic of GBM. Specifically, GAMs regulate the ability of GBM tumor cells to evade clearance by the immune system by down regulation of antigen presentation and subsequent T-cell activation (43). Importantly, elevated levels of PGE2 in the GBM-TME were demonstrated to downregulate major histocompatibility complex class II (MHC class II), responsible for antigen presentation, in microglia (44). In patients, expression of MHC class II is downregulated in GAMs isolated from patients with GBM, leading to ineffective T-cell activation and immunosuppression (45). Induction of COX-2/PGE2 leads to robust production of immunosuppressive mediators such as IL-6, IL-10, and GM-CSF that lead to induction of regulatory T cells, further exacerbating immunosuppression (44, 46). Microglial mTOR/STAT3 signaling is also upregulated in GBM, triggering immunosuppression through induced expression of IL-6 and IL-10 and inactivation of microglial mTOR (43).

Advances in the genomic landscape of the GBM TME has demonstrated the significant roles that GAMs play in tumor progression, but there is still much to be elucidated concerning GAM heterogeneity, plasticity, and classification. It has become apparent that these myeloid populations are highly dynamic, represent spatial diversity, and need to be evaluated multidimensionally. This complexity is in poorly represented by simplified M1/M2 framework that is commonly used to describe macrophage phenotypes. Classically, macrophages and microglia have been categorized through the dualistic lens of M1 (pro-inflammatory) and M2 (anti-inflammatory) activation states. In context of GBM, M1 represents an anti-tumor phenotype, while M2 is described as pro-tumor (47). Microglia being the resident brain macrophages, adopted this nomenclature as well without regard to the distinct differences between them. As research in the field advanced, it became clear that a significant amount of *in vitro* data that supported the M1/M2 framework could not be recapitulated *in vivo* (48–50). Additionally, single cell analysis revealed distinct phenotypic and spatial differences between GAMs in human GBM samples and that both M1 and M2 markers were expressed concurrently in microglia (51). The dichotomous M1/M2 system fails to reflect heterogeneity, spatial landscape, ontogeny, or disease states (52, 53). A recent review has elegantly demonstrated this new concept by presenting GAMs in spatial association to primary brain tumor type, identified potential markers that differentiate macrophages from microglia, and outlined factors that may support microglia heterogeneity in the TME (22).

## COX-2: Activity and expression

COX-1/2, also known as Prostaglandin G/H synthase 1/2 (PTGS1/2) respectively, are key rate limiting enzymes that covert arachidonic acid (AA) into prostaglandin G2 (PGG2) and PGH2 which can then be metabolized by prostaglandin E synthase (PGES) downstream to form 5 bioactive lipids known as prostanoids (16, 54). These five prostanoids are PGE2, PGI2, PGD2, PGF2a, and thromboxane A2 (TXA2). Induction of COX activity and its downstream products are linked to classic inflammatory states such as fever, acute pain, local tissue injury, and arthritis, and as such it is targeted by classic non-steroidal anti-inflammatory drugs in treating these conditions (55). While COX-1 is expressed constitutively throughout most tissues and acts a homeostatic inflammatory mediator for requisite physiological tasks, COX-2 has very low constitutive expression in most tissues, but its expression is rapidly inducible in response to pathological insults and inflammatory stimuli such as cytokines, growth factors, and various tumor promoters (16, 56). COX-2 gene expression is regulated by regulatory cis-elements in its promoter. The two most well characterized critical elements for regulation are the cAMP response element (CRE), which is recognized and activated by dimeric transcription factor activator protein 1 (AP1) and CRE binding protein (CREB), and two nuclear factor kappa B (NF-κB) consensus binding sites, which bind p65 NFκB. Additional sites include a CCAAT/enhancer, which is activated by and the CCAAT/enhancer binding protein (C/EBP). Together, these transcription factors recruit transcriptional co-activator p300 to the AP1/CREB/NFκB/C/EBP regulatory complex, and this complex is essential for proper COX-2 transcription initiation (57). Therefore, COX-2 expression is induced by multiple interacting transcription factors and their associated binding partners (57).

Diverse extracellular stimuli induce the expression of COX-2 through activation of cell surface receptors that initiate signaling cascades which culminate in the regulation of these transcription factors. Classically, lipopolysaccharide (LPS) stimulates toll-like receptor 4 (TLR4) to engage the adapter molecule myeloid differentiation factor 88 (MyD88), which then signals through Mitogen Activate Protein (MAP) kinase cascades to induce AP1 activation and association with the COX-2 promoter. The IL-1 receptor induces COX-2 expression through similar MyD88-dependent MAP kinase activation upon activation by its ligand, IL-1β (58). C/EBP is also activated downstream of MAP kinase activation. LPS/TLR4 activation also triggers MyD88-dependent activation of tumor progression locus 2 (Tpl2), which leads to nuclear translocation and activation of both NFκB and CREB. In addition to receptor-stimulated regulation, COX-2 expression can be upregulated by hypoxia, which triggers NF-κB interaction with the NF-κB regulatory element and recruitment of Hypoxia Inducible Factor 1α (HIF-1α) to the COX-2 promoter (59, 60). Finally, Nitric oxide (NO), a small molecule converted from L-arginine by inducible nitric oxide synthase (iNOS), can enhance COX-2 expression through activation of CREB (61). Therefore, COX-2 transcriptional regulation reflects convergent, integrated regulation by multiple stimuli.

## PGE2/EP2 signaling

**Figure 1:**
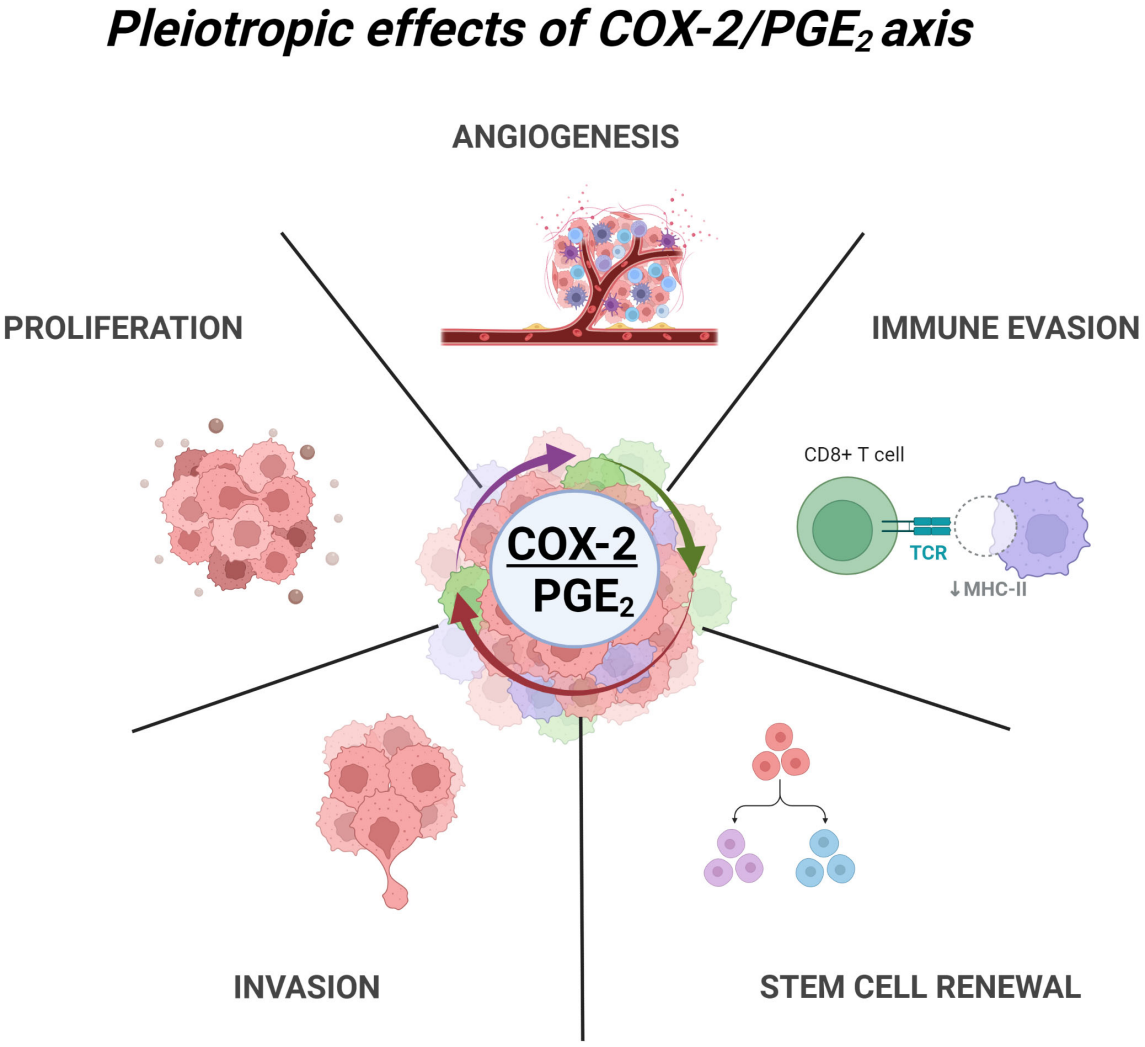
Pleiotropic effects of COX2/PGE2 axis in GBM. COX-2 dependent production of PGE2 leads to multiple tumor promoting effects through activation of EP1-4 receptors. These include angiogenesis, immune evasion, glioma stem cell renewal, invasion and ECM remodeling, and enhance proliferation. Created with BioRender.com.

The diversity of effects of COX-2/PGE2 on angiogenesis, tumor-promoting inflammation, invasion, and immunosuppression in GBM reflects the diversity of signaling pathways regulated by these mediators (**Figure 1**). PGE2 binds and signals through the EP family of receptors (EP1-4). Due to the functional variability of these receptors, PGE2 initiates pleiotropic downstream effects. EP receptors are all G-protein coupled receptors (GPCRs) with distinct downstream effects depending on their G-protein coupling. Activation of Gq-coupled EP-1 leads to activation of phospholipase C (PLC), which increases intracellular Ca^2+^ and activates protein kinase C (PKC). The EP-3 receptor is primarily Gi-coupled, resulting in inhibition of the adenylate cyclase/cAMP signaling and activation of Gβγ dependent signaling. EP-2 and EP-4 are both Gs-coupled receptors that activate cAMP formation through adenylate cyclase which leads to activation of the protein kinase A (PKA) pathway. EP2/4 activation by PGE2 leads to β-arrestin recruitment, activation of proto-oncogene tyrosine-protein kinase (c-Src), and subsequent transactivation of epidermal growth factor receptor (EGFR), initiating downstream phosphoinositide 3-kinase (PI3K)–Akt, MAPKinase, Ras/Raf, and c-Jun N-terminal kinase (JNK) pathway signaling, all known to increase cell proliferation, migration, and differentiation (62–65). A distinct difference between EP-2/4 is that, upon PGE2 activation, the EP-4 receptor becomes rapidly internalized and desensitized, while EP-2 rarely internalizes and sustains persistent receptor signaling at the cell surface (66).

PGE2 is the predominant downstream product of COX-2 and is implicated in tumor growth and progression in multiple solid malignancies such as breast (67), colorectal (68), lung (69), skin (70), pancreatic (71), prostate (69) and CNS tumors (16). In gliomas COX-2/PGE2 expression is correlated with an increase in glioma grade and poor prognosis. A study of 66 patient glioma samples revealed that 71% of GBM tumor samples had higher than 50% COX-2 positive cells (3% had less than 25% COX-2 positive cells) compared to 30% COX-2 positive cells of low-grade gliomas (40% had less than 25% COX-2 positive cells) (72). COX-2 production of PGE2 is induced upon the treatment of GBM patients with both radiation and chemotherapy leading to a steep increase of immunosuppressive cytokines (16). Elevated COX-2/PGE2 has been shown to correlate with decreased survival and earlier recurrence following radiaotherapy (14, 41). Additionally, levels of circulating PGE2 in patients were shown to decrease significantly following surgical resection of malignant tumors (73). Spatial expression of COX-2 in GBM shows that the majority of COX-2 expression is localized to the core of the tumor, dissipating in the periphery, and is negligible in adjacent tissues. This pattern of expression is consistent with the fact that GBM characteristically maintains a hypoxic microenvironment particularly in the central regions of tumor and hypoxia facilitates COX-2 upregulation in a HIF-1α dependent manner (60, 74).

**Figure 2:**
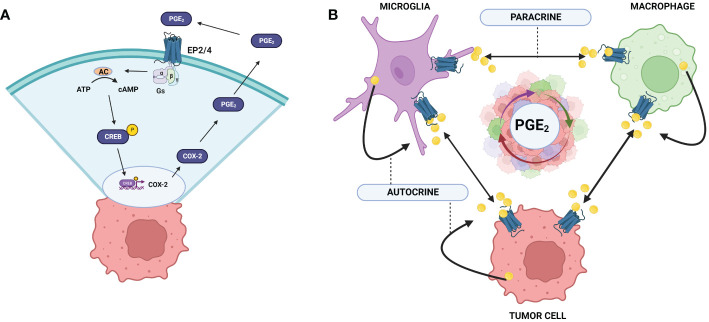
COX-2/PGE2 autocrine and paracrine feedback loops in the GBM TME. **(A)** COX-2 expression is induced through the activation of EP-2/4 by PGE2. Activation of CREB leads to association with the COX-2 promoter region and upregulation of COX-2 expression. **(B)** Tumor cells and GAMs upregulate COX-2 expression upon PGE2 activation through autocrine and paracrine mechanisms. Exacerbation of this cycle enhances robust upregulation of COX2/PGE2 in the GBM TME leading to tumor promoting effects and poor prognosis. Created with BioRender.com.

Multiple feed-back regulatory loops exist between COX-2 production of PGE2 and PGE2 regulation of COX-2 expression, amplifying the pro-tumor, immunosuppressive influences of COX-2/PGE2 on the TME (**Figure 2A**) (75–77). PGE2 stimulation of the EP2 and EP4 receptors activates nuclear translocation of CREB and binding to the COX-2 promoter, COX-2 expression, and production of more PGE2 (78). In the presence of glioma-derived soluble factors, microglia produce significant amounts PGE2, establishing a paracrine mechanism as well (12). This feedback loop may give context to the high correlation of COX-2/PGE2, as well as the percentage of infiltrating GAMs with high grade gliomas and poor prognosis. Additionally, PGE2 induces VEGF through HIF-1α activation, and VEGF can stimulate COX-2/PGE2 production, suggesting that these mediators are also co-regulated in a feed-forward, amplifying mechanism (76, 79). VEGF overexpression in the GBM-TME is associated with poor prognosis, and this PGE2/VEGF axis may contribute to the prevalence of angiogenesis and invasiveness of GBM. Macrophages, microglia, and tumor cells sustain the ability to produce and respond to COX-2/PGE2 through autocrine/paracrine signals creating a cyclical storm of inflammatory mediators (**Figure 2B**).

## Therapeutic implications

GBM is notoriously resistant to conventional therapies, driving a need for additional targets and approaches. COX-2’s multifaceted role in cancer progression suggests it may be a potential target for therapy. Inhibition of COX-2 by nonsteroidal anti-inflammatory drugs (NSAIDs) is a common treatment of cancers and it has increased patient survival in some cancers (80). However, NSAIDs are not selective for COX-2; they also target COX-1 and the related side-effects, including upper gastrointestinal (GI) stress, limit their use (81). The development of COX-2 selective inhibitors (COXIBs) in the late 1990s was met with major enthusiasm and great expectations for safer COX-2 inhibition. However, while these drugs do indeed display lower GI stress, the initial enthusiasm for their use has been dampened by significant cardio- and cerebro-vascular toxicities (82). COX-2 selective inhibitors have shown some efficacy in clinical trials as an adjuvant to chemotherapy and radiotherapy (16, 81). Therefore, even though there is clear evidence that COX-2 function is a plausible target in the treatment of GBM, direct inhibition of the enzyme with selective inhibitors may not be an effective strategy.

The multifaceted physiological roles of COX-2 limit its potential as a direct target for long-term therapeutic use. However, therapeutic intervention targeting the cyclical upregulation of COX-2/PGE_2_ in the TME can be achieved without direct COX-2 inhibition, and these indirect strategies may provide safety and efficacy advantages. A promising approach is targeting of downstream mediators of COX-2, especially PGE2, and their receptors. The pleiotropic effects of autocrine and paracrine signaling through the COX-2/PGE2 axis in the tumor microenvironment need to be further delineated to target specific paths that lead to malignant progression. Isolating the specific effects of COX-2/PGE_2_ for individual EP receptors and how they each shape GBM TME in a spatial and temporal manner will inform future therapeutic avenues. For example, PGE_2_/EP_2_ signals through a G protein-dependent pathway (cAMP/CREB) and PGE_2_ stimulates VEGF production through multiple mechanisms (HIF-1α activation, cAMP signaling, and EGFR transactivation) promoting angiogenesis (76). The essential role of the EP_2_ receptor in the autocrine/paracrine signaling establish it as an attractive target for intervention. In recent years, multiple EP_2_ small molecule inhibitors have been identified and tested, including butaprost, CAY10399, ONO-AE1–259, and TG6‐10‐1 (83). The brain-permeable, small molecule EP_2_ antagonist TG6‐10‐1 has shown early promise as a possible therapeutic. In a recent study, inhibition of the PGE_2_/EP_2_ signal cascade by TG6‐10‐1 demonstrated significantly reduced GBM tumor growth in both subcutaneous and intercranial *in vivo* models (84).

While there has been extensive research into COX- in inflammation and cancer, the specifics of its dynamic regulation within and among the diverse cell types in the TME has yet to be fully elucidated. Understanding how induction of COX-2 expression is regulated in the context of GBM-TME may reveal therapeutic targets and strategies that are more selective than global COX-2 inhibition. For example, RGS10, a small G-protein regulator, has been shown to be a robust regulator of COX-2/PGE2 in both macrophages and microglia. RGS10 strongly suppresses COX-2 following activation by diverse upstream activators, including LPS, TNFα, and interferon gamma (85, 86). RGS10 does not completely abrogate COX-2, but attenuates the stimulated induction of COX-2 expression in stimulated cells (87). Therefore, RGS10 represents a potential target to break the cycle of COX-2 expression and PGE-2 production in GBM (86).

## Concluding remarks

The GBM microenvironment is a dynamic system, and its high heterogeneity leads to an immunosuppressive environment. Tumor cells recruit immune cells which aid in this immunosuppression through production of inflammatory mediators. Infiltration of GAMs leads to dysregulated inflammatory states that promote tumor progression. COX-2 and PGE2 are increased in GBM, and their pleiotropic signals impact proliferation, angiogenesis, immune evasion, stem cell renewal, and invasion. GBM lacks an effective treatment strategy. Harnessing the COX-2/PGE2 axis and understanding GBM microenvironment dynamics are important steps to revealing potential targets and informing new therapeutics.

## Author contributions

SH and PD were responsible for the design, writing, and editing of the manuscript. All authors contributed to the article and approved the submitted version.
